# Evolving dimensions in medical case reporting

**DOI:** 10.1186/1752-1947-5-164

**Published:** 2011-04-27

**Authors:** Aristotle D Protopapas, Thanos Athanasiou

**Affiliations:** 1 28 Old Brompton Road, London, SW7 3SS, UK; 2 Division of Surgery and Cancer, Imperial College London, QEQM Wing, St Mary's Hospital, Praed Street, London, W2 1NY, UK

## Abstract

Medical case reports (MCRs) have been undervalued in the literature to date. It seems that while case series emphasize what is probable, case reports describe what is possible and what can go wrong. MCRs transfer medical knowledge and act as educational tools. We outline evolving aspects of the MCR in current practice.

## 

The full translational potential of medical case reports (MCRs) is not always considered by authors, periodicals or readers, as MCRs are often perceived as a low-budget form of publication for fledgling medical writers. The acceptance rate for MCRs and their priority for publication are lower than those for other manuscripts in traditional journals. It is important to emphasize that prospective, retrospective and observational randomized controlled trials are always constructed on the basis of data obtained from individual patients whose cases are the units that create the cohort, allowing the investigator to define end points and make inferences by calculating effect sizes. It is safe to say that all classes of evidence (Classes I through III) are constructed using the accumulated units of observation comprising individual cases. Although MCRs are limited by the fact that they cannot be generalized beyond the context of the individual patient or patients described [1] and thus are not suitable for inference, they offer a high degree of opportunity to transfer medical knowledge and act as educational tools, and in a very direct way.

In this editorial, we attempt to outline the evolving dimensions of MCRs in four particular areas of medical education: (1) reporting of adverse events (AEs), (2) new diseases or exceptional environments, (3) medical innovation and (4) appropriate use of media in terms of ethics, standardization and creativity (Figure 1).

**Figure 1:**
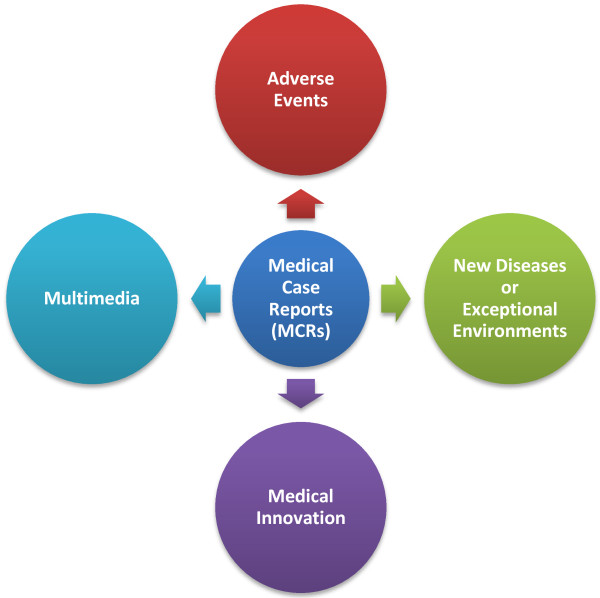
Evolving dimensions increasing the educational value of MCRs

## MCRs of adverse events: errors of omission or errors of commission?

An AE is an unwanted event that occurs in the course of treatment, especially in a clinical trial. MCRs may be the first warning of catastrophic AEs [2,3].

The *Journal of Medical Case Reports *encourages constructive MCRs of AEs, especially the unreported side effects or adverse interactions involving medications or unexpected events in the course of treating a patient [4].

An isolated AE can be important in forming part of the evidence in the healthcare sciences [5]. Some methodologies of randomized controlled trials lead to the exclusion of such isolated AEs from their data sets, which renders an isolated MCR of an AE even more valuable.

It is especially crucial to highlight translational AEs, where *in vitro *or animal experiments have not been reproduced in humans, with resultant ramifications for patient safety [6]. The evidence in national and international AE databases should be consulted, and each AE should be logged appropriately [7].

Overall, an AE should be reported in an exacting, scientific way. A root cause analysis should be included, and a survey of evidence-based recommendations should conclude the report. Below we present a brief algorithm from the generic template of *JMCR*[4] that summarizes the above-mentioned contentions:

1. Introduction: The Introduction should state the indications of the intervention, a brief overview of existing evidence and current evidence-based recommendations, preferably tabulated and classified [8].

2. Case presentation: Are case presentations an error of omission or errors of commission? Adherence to recommendations and good practice are essential.

3. Discussion: The Discussion section should translate findings to clinical best practices and patient safety. In cases in which a case report describes an AE that occurred within the setting of a randomized controlled trial, this should be clearly stated and details of the randomized controlled trial, such as its registration and relevant reference of the protocol, should be included in the MCR.

4. Conclusion: The Conclusion section should comprise a root cause analysis.

## MCRs of new diseases, rare conditions or exceptional environments

Case reports remain an important source of evidence for rare conditions or exceptional treatment environments. Large trials are not possible in such cases, and MCRs offer important treatment information. Typical examples include the description of a new or previously undescribed genetic condition with an atypical inheritance pattern or the management circumstances of individual patients in the context of geographic or physiological extremes (including high altitude and major disasters). In the absence of larger data sets, these individual cases offer valuable information for healthcare practitioners in treating any similar patients when they occur.

## MCRs of innovation

We feel that case reports describing innovative techniques advance healthcare and biotechnology by translating, validating and finally returning data from individual patients for further development and refinement of technologies with a low cost-impact ratio. The major issue of biomedical innovation is patient safety, and this should be reflected in reporting a novel intervention. The relevant institutional, national and international guidelines should provide benchmarks for innovations and should be adopted in drafting the MCR manuscript.

## Appropriate use of media for MCRs: ethics, standardization and creativity

The expanding role of multi-media in driving home a message from a case presentation and attracting readership cannot be overemphasized. *JMCR *aspires to describe ethical, high-quality imaging modalities in MCRs.

## Ethics

*JMCR*'s mandatory policy on consent to publish [9] applies especially to the explicit consent of the reported patients to have their images, X-rays and histological films published. It is also important to keep in mind that the author guidelines of *JMCR *state that authors must preserve the anonymity of the patient [4]. It is expected that all photographs of humans and reproductions of medical imaging (for example, computed tomographic (CT) scan slices) are stripped of any identifying information. The free and open access to *JMCR *articles renders these precautions even more important, as the general public has access to every picture!

## Standardization

A small, selective study [10] found a lack of standardization and relevance presented in published radiological images. It is important that the legend of each image be accurate and directly relevant to the MCR.

## Creativity

Media, being a direct non-verbal message *per se*, should display creativity in our current Information Age. Digital photography is currently accessible to most healthcare organizations around the world, facilitating the capture and transmission (that is, uploading) of visual data. The use of medical photography expertise is a sound investment, yet conventional medical illustrators need to evolve and diversify from sketching to digitized multi-media (that is, platform non-specific and viewable using free or widely available tools [4,11]).

The time has come for streaming media to replace traditional forms of media. An example is making whole CT sequences (axial or three-dimensional), as opposed to still, selected slices, available in the MCR [11].

## Summary

In this era of digitization, case reporting necessitates a shift of gravitas to patient safety, the application of improved multi-media and an overall increase in educational potential. In this brief editorial, we have attempted to guide prospective authors of MCRs in optimizing their creative writing from ethical and practical points of view, especially with regard to patient safety. This guidance is meant to complement *JMCR*'s general instructions to authors [4].

## Competing interests

The authors declare that they have no competing interests.

## Authors' contributions

ADP and TA were equal contributors in writing the manuscript. We both read and approved the final manuscript.
